# Controlled Heel‐Site Arteriovenous Vent via Venous Reimplantation on PTFE‐Tibial Bypass: A Novel Adjunct in No‐Vein Chronic Limb‐Threatening Ischemia

**DOI:** 10.1155/carm/2506957

**Published:** 2026-09-15

**Authors:** Francesco Spinelli, Nunzio Montelione, Mario Malangone, Vincenzo Catanese, Julia Paolini, Francesco Stilo

**Affiliations:** ^1^ Vascular Surgery Unit, Fondazione Policlinico Campus Bio-Medico, Rome, Italy, unicampus.it; ^2^ Research Unit of Vascular Surgery, Department of Medicine and Surgery, Università Campus Bio-Medico di Roma, Rome, Italy, unicampus.it

**Keywords:** case report, chronic limb-threatening ischemia, distal arteriovenous fistula, PTFE tibial bypass, venous reimplantation

## Abstract

**Background:**

In no‐vein chronic limb‐threatening ischemia (CLTI), infrapopliteal prosthetic bypass remains a limb‐salvage option but is limited by high distal resistance and graft‐artery compliance mismatch. Standard distal adjuncts, including vein cuffs, patches, or distal arteriovenous fistulas, may be technically demanding when local tibial venous anatomy and the confined distal exposure do not allow a reliable conventional configuration. We describe a compact heel‐site bypass‐to‐vein vent based on direct end‐to‐side venous reimplantation onto the PTFE graft immediately proximal to the arterial heel.

**Case Presentation:**

A controlled heel‐site arteriovenous vent was created immediately proximal to the arterial heel of a distal polytetrafluoroethylene (PTFE)–anterior tibial artery (ATA) anastomosis in a 75‐year‐old man with recurrent CLTI, prior failed revascularizations, no usable autologous conduit, and single‐vessel tibial runoff through the ATA. Intraoperative assessment showed paired deep veins accompanying the ATA of approximately 2.0–2.5 mm in external diameter with thin walls, making a conventional distal AVF/interposition configuration technically unfavorable. A calibrated oval venotomy was fashioned on the distal PTFE graft, and the proximal stump of the anterior tibial vein was reimplanted end‐to‐side onto the graft with an oblique takeoff to create a short, controllable bypass‐to‐vein vent.

**Results:**

Postoperative computed‐tomography angiography and serial duplex ultrasound surveillance demonstrated sustained patency of both the PTFE–ATA bypass and the heel‐site vent, without hemodynamically significant stenosis, excessive shunting, venous hypertension, distal steal, graft thrombosis, or reintervention at 6 months. Rest pain resolved, and baseline ambulation was restored.

**Conclusions:**

This heel‐site arteriovenous vent represents a compact, calibratable bypass‐to‐vein configuration that differs from conventional distal AVF adjuncts by using direct end‐to‐side venous reimplantation onto the PTFE graft immediately proximal to the arterial heel. Because established distal AVF and vein‐cuff adjuncts are supported by a stronger evidence base, this configuration should be considered only as a fallback/adaptive option in selected no‐vein CLTI scenarios when standard distal adjuncts are technically infeasible.

## 1. Introduction

Chronic limb‐threatening ischemia (CLTI) represents the most severe clinical spectrum of peripheral artery disease and is associated with high morbidity, mortality, and limb loss [1]. Evidence‐based guidelines advocate prompt revascularization, and autogenous vein bypass remains the preferred option in patients with advanced limb threat or complex anatomy [1]. However, a substantial proportion of CLTI patients lack an adequate autologous conduit for bypass, and infrapopliteal targets are frequently small, calcified, and heavily diseased [2]. In these circumstances, ringed polytetrafluoroethylene (PTFE) grafts may be used for limb salvage, but their patency is inferior to that of autogenous vein conduits, particularly for tibial targets [3].

Several distal adjuncts have been developed to mitigate the hemodynamic limitations of prosthetic infrapopliteal bypass. Vein cuffs or patches, such as the Miller cuff, enlarge the distal suture line and improve compliance matching between the prosthetic graft and the native artery [3, 4]. Another strategy is the creation of a distal arteriovenous fistula (AVF), which provides a low‐resistance outflow pathway to augment graft flow [4]. Ascer and colleagues described a combined deep‐vein interposition and distal AVF technique that achieved favorable assisted primary patency in complex infrapopliteal reconstructions [4]. Similar concepts have been supported by long‐term experience with venous interposition AVFs in no‐vein patients undergoing below‐the‐knee prosthetic bypass [2, 5, 6].

Despite these potential advantages, distal AVF adjuncts require careful calibration. Excessive shunting may cause distal steal or require immediate banding, whereas inadequate shunt flow may fail to improve graft hemodynamics [4, 7]. Moreover, Ascer‐type distal AVF/interposition configurations rely on the paired deep veins accompanying the tibial arteries; when these veins are of small caliber or poor wall quality, such configurations may be technically unfavorable. The present case describes a controlled heel‐site arteriovenous vent created by direct end‐to‐side reimplantation of the anterior tibial vein onto a small calibrated opening on the distal PTFE graft immediately proximal to the heel of a PTFE–anterior tibial artery (ATA) anastomosis, as a compact fallback/adaptive bypass‐to‐vein configuration for a selected no‐vein CLTI scenario.

## 2. Case Presentation

A 75‐year‐old man with CLTI of the right lower limb and a history of multiple revascularizations presented with progressive rest pain and nonhealing forefoot wounds despite optimal medical therapy. Prior limb revascularization consisted of an autogenous superficial femoral–posterior tibial bypass. Approximately 2 months later, limited forefoot amputations were performed for tissue loss. The bypass remained clinically functional for approximately 39 months, after which recurrent ischemic symptoms prompted repeat imaging that demonstrated distal outflow failure and occlusion. This was managed with surgical revision and an additional short venous jump graft to the distal posterior tibial/plantar segment. Symptom relief was transient, and approximately 5 months later, the patient represented with recurrent CLTI, leading to the index prosthetic infrapopliteal bypass described below.

**FIGURE 1:**
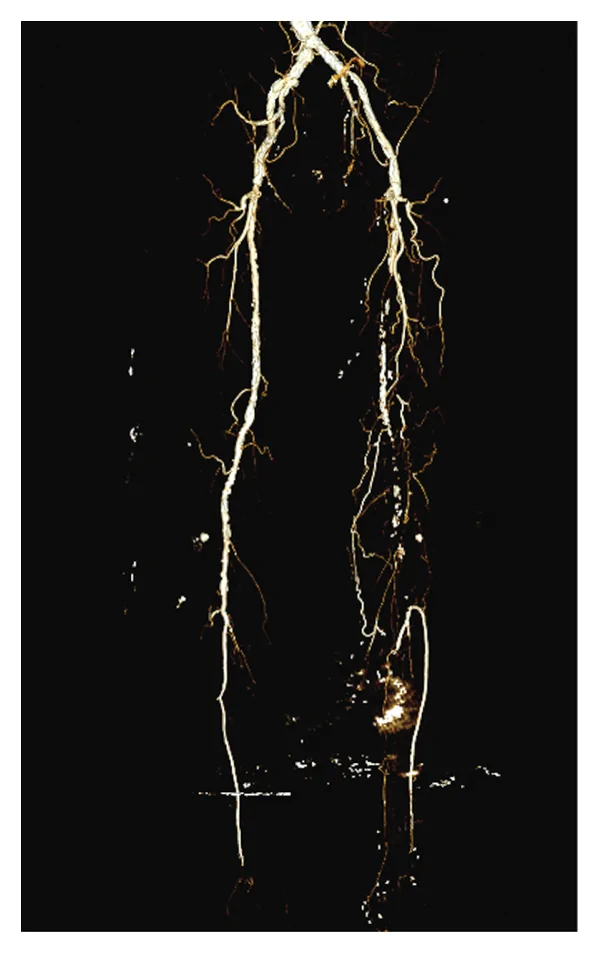
Preoperative computed‐tomography angiography demonstrating complex femoropopliteal and prior bypass occlusive disease with severely compromised tibial runoff. The native superficial femoral artery and a previous femorotibial bypass are occluded, with associated popliteal occlusion; the ATA is the only reliable outflow vessel, supporting the indication for prosthetic infrapopliteal bypass with a planned distal adjunct.

Preoperative computed‐tomography angiography demonstrated occlusion of the native superficial femoral artery and the previous femorotibial bypass, popliteal occlusion, and severely compromised tibial runoff, with the ATA as the only reliable outflow vessel (Figure 1). In the absence of usable autologous conduit, prosthetic infrapopliteal bypass with a planned distal adjunct was selected, with intraoperative assessment of whether a conventional Ascer‐type distal AVF/interposition configuration was technically feasible.

Under general anesthesia, the common femoral artery and distal ATA were exposed through limited incisions. Systemic heparinization was administered with 5000 IU of unfractionated heparin, and atraumatic vascular control was obtained. A 6‐mm ringed PTFE graft was anastomosed end‐to‐side to the common femoral artery using fine monofilament suture. The conduit was routed to the distal field in the suprafascial plane along the lateral aspect of the thigh and into the anterior compartment of the leg, reaching the distal ATA between the tibialis anterior and extensor hallucis longus muscles.

**FIGURE 2:**
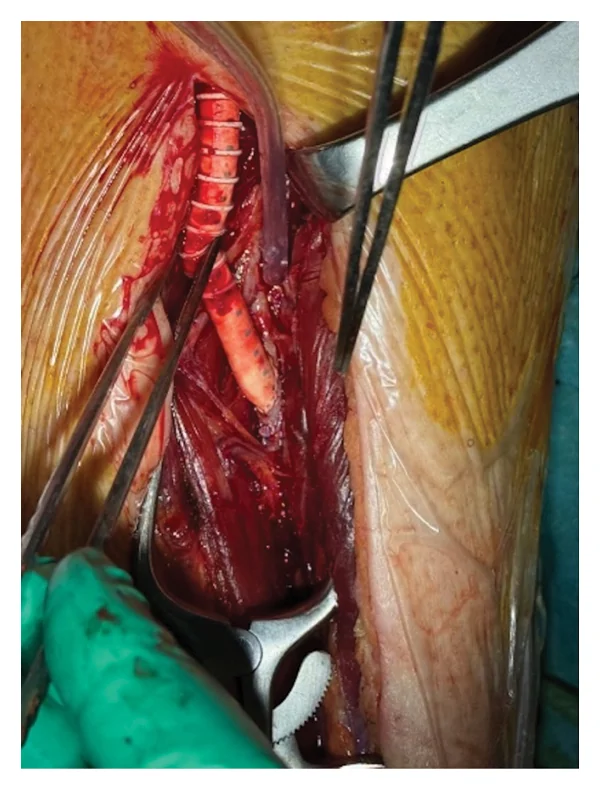
Intraoperative view of the controlled heel‐site arteriovenous vent during distal ringed PTFE–anterior tibial artery (ATA) reconstruction. After exposure of the ATA and an accompanying anterior tibial vein, a small calibrated oval venotomy is created on the distal PTFE graft immediately proximal to the arterial heel. The anterior tibial vein is transected, its distal end ligated, and the proximal stump anastomosed end‐to‐side to the graft in an oblique, approximately 30–45‐degree orientation, creating a short, controlled bypass‐to‐vein vent before completion of the distal graft‐to‐ATA anastomosis.

**FIGURE 3:**
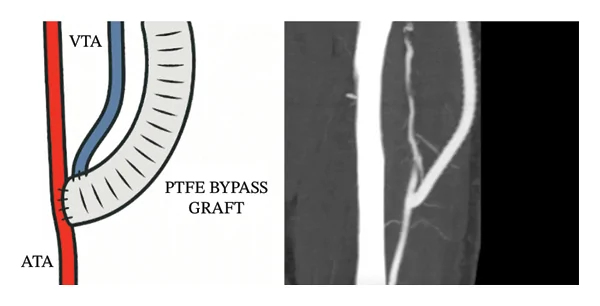
Schematic and postoperative imaging representation of the distal reconstruction. Left: ringed PTFE–ATA end‐to‐side anastomosis with the controlled heel‐site bypass‐to‐vein vent obtained by direct end‐to‐side reimplantation of the anterior tibial vein onto a small calibrated oval venotomy on the distal PTFE graft just proximal to the arterial heel; the oblique venous takeoff is designed to streamline shunt flow and limit turbulence. Right: postoperative vascular imaging demonstrating patency of the distal reconstruction.

A venous interposition AVF following the technique described by Ascer was initially considered [4]. At direct exploration, the paired deep veins accompanying the ATA were approximately 2.0–2.5 mm in external diameter and thin walled, making a conventional distal AVF/interposition configuration technically unfavorable and leading to an intraoperative fallback strategy. To avoid a simple graft‐to‐artery anastomosis in a high‐risk outflow setting, a controlled heel‐site bypass‐to‐vein vent was created (Figures 2 and 3). After approximately 2 cm of the ATA and an accompanying anterior tibial vein had been freed from the surrounding tissues, the PTFE graft was cut obliquely to create an elliptical distal end for the arterial end‐to‐side anastomosis. A 2‐mm oval venotomy was fashioned on the deep aspect of the graft, 1‐2 mm proximal to the proximal end of the ellipse. The anterior tibial vein was mobilized and sectioned obliquely; its distal end was ligated, and the proximal stump was anastomosed end‐to‐side to the graft opening with continuous 8‐0 monofilament suture. The venous takeoff was oriented obliquely, approximately 30–45° to the graft axis, to streamline shunt flow and minimize turbulence adjacent to the heel.

The venous ostium was deliberately limited to the native vein caliber, thereby creating a short, controllable bypass‐to‐vein vent rather than a high‐flow fistula. The ATA was then opened, and the arteriotomy length was tailored to approximately 1.2–1.5 times the graft diameter to improve compliance matching. The distal graft‐to‐ATA anastomosis was completed with 7‐0 monofilament suture in standard fashion. Before completion, the system was flushed and deaired. Completion angiography and duplex interrogation confirmed brisk conduit flow along the PTFE graft and across the distal anastomosis, preserved pedal signals, and no hemodynamic evidence of distal steal. No immediate technical complications occurred.

Early postoperative computed‐tomography angiography demonstrated wide patency of the PTFE–ATA bypass, uniform graft opacification, absence of kinking, and brisk runoff through the distal ATA (Figure 3). The heel‐site arteriovenous vent was opacified as a small‐caliber, low‐resistance channel without thrombus, stenosis, contrast extravasation, or pseudoaneurysm. Three‐dimensional volume‐rendered reconstructions confirmed reestablishment of flow through the reconstruction and into the distal tibiopedal circulation.

The immediate postoperative course was uneventful. Analgesia was managed with scheduled acetaminophen and adjunct pregabalin, without opioid requirement beyond the first 24 h. Postoperative antithrombotic therapy consisted of low‐molecular‐weight heparin 4000 IU once daily for 6 days, together with aspirin 100 mg and clopidogrel 75 mg daily. Dual antiplatelet therapy was continued for 3 months, after which single antiplatelet therapy was maintained. High‐intensity statin therapy and standard gastroprotection were continued.

Rest pain decreased progressively during the first postoperative week and had fully resolved at the 2 week control. Surgical wounds remained clean and dry, without dehiscence or signs of infection. The limb remained warm and well perfused, with preserved pedal signals and no edema suggestive of venous hypertension. No clinical features of distal steal were observed.

**FIGURE 4:**
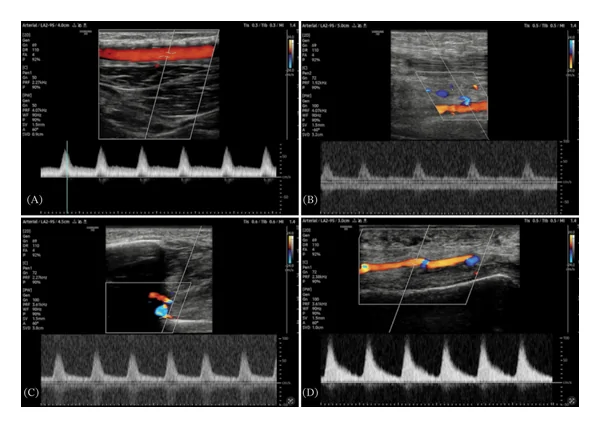
Representative duplex ultrasound surveillance showing sustained patency of the PTFE–ATA bypass and the controlled heel‐site bypass‐to‐vein vent without hemodynamically significant stenosis or excessive shunting. Labels: (A) PTFE graft; (B) anterior tibial vein takeoff/controlled heel‐site bypass‐to‐vein vent; (C) proximal ATA; and (D) dorsalis pedis artery.

A duplex‐based surveillance protocol was followed at 2 weeks and at 1, 3, and 6 months (Figure 4). At each assessment, the PTFE–ATA bypass was patent, with multiphasic forward flow in the distal ATA and stable velocities throughout the graft. The heel‐site arteriovenous vent remained patent and functioned as a low‐resistance communication without hemodynamic evidence of excessive shunting. No focal peak systolic velocity acceleration or elevated velocity ratio suggestive of hemodynamically significant stenosis was detected at the heel or toe of the distal anastomosis.

No reinterventions were required, and there were no episodes of graft thrombosis, distal steal, or venous hypertension. The patient reported durable relief of rest pain and recovery of baseline ambulatory function by the 3‐month control, sustained at 6 months. A formal patient‐perspective statement was not collected.

## 3. Discussion

In patients without adequate saphenous vein, below‐the‐knee prosthetic bypass may be the only available option for limb salvage, but early failure remains a major concern. Synthetic grafts to small‐caliber tibial arteries create high distal resistance and a compliance mismatch at the distal anastomosis, both of which promote disturbed flow and thrombosis [5, 8]. Vein cuffs, collars, and patches are used to smooth the prosthetic‐to‐arterial transition and improve compliance matching [8–10]. Distal AVFs, as popularized by Ascer, lower effective outflow resistance and augment graft flow [4, 11].

Established distal AVF adjuncts, including Ascer‐type configurations, use the paired deep veins accompanying the tibial arteries. In the present report, the distinction lies in the geometry of the reconstruction.

The reconstruction consisted of a ringed PTFE bypass to the ATA with a standard end‐to‐side distal arterial anastomosis, combined with direct end‐to‐side reimplantation of the proximal anterior tibial vein stump onto a small calibrated oval opening on the distal PTFE graft a few millimeters proximal to the arterial heel. Thus, the venous limb was not used as an interposition segment, cuff, patch, or classic distal fistula incorporated into the arterial suture line. The resulting vent was compact, independently calibratable, and anatomically separate from the graft‐to‐ATA anastomosis, with preservation of the arterial toe and antegrade tibiopedal runoff. The venous limb could theoretically be tightened, banded, or ligated if excessive shunting occurred.

The pathophysiologic rationale is twofold. First, the heel‐site vent provides a low‐resistance parallel outflow pathway that increases mean conduit throughput, improves time‐averaged wall shear stress, and reduces stagnation within the PTFE graft, all of which are relevant to early thrombosis in small‐caliber tibial targets. Second, the venous limb may behave as a localized compliant reservoir, partially reproducing a Windkessel‐like effect and tempering graft‐artery compliance mismatch at the distal suture line [12]. Because the shunt is short and deliberately small, antegrade tibiopedal perfusion is intended to be preserved while the risk of distal steal is minimized. These potential advantages are hypothesis‐generating and should not be interpreted as evidence of superiority over established distal AVF or vein‐cuff techniques. Indeed, established distal AVF configurations and vein‐cuff adjuncts remain preferable when feasible because they are supported by a stronger evidence base [9, 11, 13]; in the present case, the heel‐site bypass‐to‐vein vent was used as a fallback/adaptive option dictated by the small caliber and thin walls of the paired deep veins accompanying the ATA.

Previous publications from the present group document extensive experience with conventional Ascer‐type venous interposition AVFs and support their use when the paired deep‐vein anatomy is suitable [5, 6]. In the present case, direct exploration showed 2.0–2.5 mm, thin‐walled paired deep veins, making conventional interposition technically unfavorable and prompting an adaptive heel‐site strategy. No validated procedure‐specific diameter threshold exists for Ascer‐type AVF/interposition. Nevertheless, in a study co‐authored by Ascer, a minimum external vein diameter below 3.0 mm was associated with poorer patency in infrapopliteal reversed greater saphenous vein bypasses; this evidence concerns saphenous conduits rather than paired deep veins [14]. In our experience, paired deep veins of approximately 3 mm or greater, with satisfactory wall quality, sufficient mobilizable length, and geometry permitting a tension‐free reconstruction without kinking, favor a conventional Ascer‐type configuration; markedly smaller, thin‐walled, or, otherwise, unfavorable veins may justify the heel‐site vent as a fallback option.

Alternative adjuncts could have included a Miller cuff or Tyrrell boot. However, these techniques require adequate perivascular space, and the confined interval between the tibialis anterior and extensor hallucis longus muscles may compress a bulkier venous adjunct, thereby limiting its compliance benefit. A distal vein patch, as promoted by Neville and colleagues, could also have been considered, but it does not provide the same direct flow‐augmentation effect as an arteriovenous communication [10].

At 6‐month follow‐up, both the prosthetic bypass and the heel‐site arteriovenous vent remained patent, rest pain had resolved, and no distal steal, venous hypertension, tissue loss progression, or reintervention occurred. These findings are consistent with the intended hemodynamic effect of graft‐flow support without clinically relevant diversion from the distal arterial bed. Prior physiologic work has shown that distal AVF creation can improve microcirculatory hemodynamics after prosthetic bypass in secondary limb salvage procedures [15]. Comparative series and systematic reviews have also recognized venous cuffs and distal AVFs among the principal adjunctive techniques designed to improve infragenicular prosthetic bypass performance [9, 11, 13].

This report has important limitations. It describes a single technical experience with short‐term follow‐up, and no direct comparison with established distal adjuncts is possible. Hemodynamic thresholds for optimal shunt calibration remain undefined, and the durability of the vent beyond 6 months requires further evaluation. Nevertheless, in a highly selected no‐vein CLTI scenario in which the paired deep veins accompanying the ATA were too small and thin walled for a conventional distal AVF/interposition configuration, the described heel‐site bypass‐to‐vein vent represents a fallback anatomic adaptation of established principles: lowering effective outflow resistance, improving flow across a prosthetic tibial bypass, and preserving antegrade tibiopedal perfusion.

## 4. Conclusion

The controlled heel‐site arteriovenous vent is a compact venous reimplantation adjunct for prosthetic tibial bypass in selected no‐vein CLTI scenarios. Unlike conventional distal AVF adjuncts based on venous interposition or fistula construction at the distal anastomotic site, this configuration uses direct end‐to‐side reimplantation of the proximal anterior tibial vein stump onto a calibrated opening of the PTFE graft immediately proximal to the arterial heel. Because established distal AVF and vein‐cuff adjuncts remain better supported by the available evidence, they should be considered preferable whenever technically feasible. The present configuration should, therefore, be regarded as a fallback/adaptive option when those conventional adjuncts are not technically feasible, as in this case because of the small caliber and thin walls of the paired deep veins accompanying the ATA. The construct couples a ringed PTFE–ATA end‐to‐side anastomosis with a short, controlled bypass‐to‐vein vent while preserving antegrade tibiopedal perfusion. In this case, early computed‐tomography angiography and serial duplex surveillance to 6 months confirmed patency of both the bypass and the vent, without steal, venous hypertension, graft thrombosis, or reintervention and with complete resolution of rest pain. Further experience is required to define reproducibility, calibration criteria, and long‐term durability.

## Author Contributions

All authors made substantial contributions to the conception or design of the work, acquisition and interpretation of clinical data, and drafting or critical revision of the manuscript and agree to be accountable for all aspects of the work.

## Funding

This research received no specific grant from any funding agency in the public, commercial, or not‐for‐profit sectors.

Open access publishing facilitated by Universita Campus Bio‐Medico di Roma, as part of the Wiley ‐ CRUI‐CARE agreement.

## Disclosure

Preliminary data from this case report have not been presented elsewhere. All authors approved the final version of the manuscript.

## Ethics Statement

The case report was conducted in accordance with the principles of the Declaration of Helsinki.

## Consent

Written informed consent for publication of the case details and accompanying images was obtained from the patient.

## Conflicts of Interest

The authors declare no conflicts of interest.

## Data Availability

The data that support the findings of this study are available on request from the corresponding author. The data are not publicly available due to privacy or ethical restrictions.

## References

[1] Conte M. S. , Bradbury A. W. , Kolh P. et al., Global Vascular Guidelines on the Management of Chronic Limb-Threatening Ischemia, Journal of Vascular Surgery. (2019) 69, no. 6S, 3S–125S.e40, 10.1016/j.jvs.2019.02.016. 31159978 PMC8365864

[2] Hingorani A. P. , Ascher E. , Marks N. A. et al., A 10 Year Experience With Complementary Distal Arteriovenous Fistula and Deep Vein Interposition for Infrapopliteal Prosthetic Bypasses, Vascular and Endovascular Surgery. (2005) 39, no. 5, 401–409, 10.1177/153857440503900504. 16193212

[3] Lauterbach S. R. , Torres G. A. , Andros G. , and Oblath R. W. , Infragenicular Polytetrafluoroethylene Bypass With Distal Vein Cuffs for Limb Salvage: A Contemporary Series, Archives of surgery. (2005) 140, no. 5, 487–494, 10.1001/archsurg.140.5.487. 15897445

[4] Ascer E. , Gennaro M. , Pollina R. M. et al., Complementary Distal Arteriovenous Fistula and Deep Vein Interposition: A Five-Year Experience With a New Technique to Improve Infrapopliteal Prosthetic Bypass Patency, Journal of Vascular Surgery. (1996) 24, no. 1, 134–143, 10.1016/s0741-5214(96)70154-4. 8691516

[5] Spinelli F. , Roscitano G. , Barillà D. et al., Long-Term Results of Below-the-Knee Bypass Using a Prosthetic Graft With a Distal Arteriovenous Fistula Interposition, Diagnostics. (2023) 13, no. 7, 10.3390/diagnostics13071246. PMC10093735 37046465

[6] Spinelli F. , D′Alfonso M. , Mandolfino T. , Acri I. , La Spada M. , and Mirenda F. , Rivascolarizzazione Distale Degli Arti Inferiori Con Protesi E FAV per Interposizione Venosa Secondo Ascer, Giornale Italiano di Chirurgia Vascolare. (2001) 8, no. 4, 285–296.

[7] Syrek J. R. , Calligaro K. D. , Dougherty M. J. , Raviola C. A. , Rua I. , and DeLaurentis D. A. , Do Distal Arteriovenous Fistulae Improve Patency Rates of Prosthetic Infrapopliteal Arterial Bypasses?, Annals of Vascular Surgery. (1998) 12, no. 2, 148–152, 10.1007/s100169900132. 9514233

[8] Tiwari A. , Cheng K. S. , Salacinski H. , Hamilton G. , and Seifalian A. M. , Improving the Patency of Vascular Bypass Grafts: The Role of Suture Materials and Surgical Techniques on Reducing Anastomotic Compliance Mismatch, European Journal of Vascular and Endovascular Surgery. (2003) 25, no. 4, 287–295, 10.1053/ejvs.2002.1810. 12651165

[9] Khalil A. A. , Boyd A. , and Griffiths G. , Interposition Vein Cuff for Infragenicular Prosthetic Bypass Graft, Cochrane Database of Systematic Reviews. (2012) 2012, no. 9, 10.1002/14651858.CD007921.pub2. PMC11569884 22972115

[10] Neville R. F. , Tempesta B. , and Sidawy A. N. , Tibial Bypass for Limb Salvage Using Polytetrafluoroethylene and a Distal Vein Patch, Journal of Vascular Surgery. (2001) 33, no. 2, 266–271, 10.1067/mva.2001.113131. 11174777

[11] Aherne T. , Kheirelseid E. , O’Neill D. et al., The Use of Arteriovenous Fistulae as an Adjunct to Peripheral Arterial Bypass: A Systematic Review and Meta-Analysis, European Journal of Vascular and Endovascular Surgery. (2016) 51, no. 5, 707–717, 10.1016/j.ejvs.2016.01.014. 27067191

[12] Westerhof N. , Lankhaar J. W. , and Westerhof B. E. , The Arterial Windkessel, Medical and Biological Engineering and Computing. (2009) 47, no. 2, 131–141, 10.1007/s11517-008-0359-2. 18543011

[13] Kreienberg P. B. , Darling R. C. , Chang B. B. , Paty P. S. , Lloyd W. E. , and Shah D. M. , Adjunctive Techniques to Improve Patency of Distal Prosthetic Bypass Grafts: Polytetrafluoroethylene With Remote Arteriovenous Fistulae Versus Vein Cuffs, Journal of Vascular Surgery. (2000) 31, no. 4, 696–701, 10.1067/mva.2000.104597. 10753277

[14] Wengerter K. R. , Veith F. J. , Gupta S. K. , Ascer E. , and Rivers S. P. , Influence of Vein Size (Diameter) on Infrapopliteal Reversed Vein Graft Patency, Journal of Vascular Surgery. (1990) 11, no. 4, 525–531, 10.1067/mva.1990.18327. 2325213

[15] Jacobs M. J. , Reul G. J. , Gregoric I. D. et al., Creation of a Distal Arteriovenous Fistula Improves Microcirculatory Hemodynamics of Prosthetic Graft Bypass in Secondary Limb Salvage Procedures, Journal of Vascular Surgery. (1993) 18, no. 1, 1–9, 10.1067/mva.1993.41521. 8326649

